# Studies of Mast Cells: Adventures in Serendipity

**DOI:** 10.3389/fimmu.2018.00520

**Published:** 2018-03-13

**Authors:** Melissa A. Brown

**Affiliations:** ^1^Department of Microbiology and Immunology, Northwestern University Feinberg School of Medicine, Chicago, IL, United States

**Keywords:** IL-4, mast cells, experimental autoimmune encephalomyelitis/multiple sclerosis, sex-dependent response, IL-33/ST2, testosterone

## Abstract

Like many of us who had the great fortune to work with Bill Paul, my science life was immeasurably altered by my interactions with him. Although intimidating at first because of his stature in the immunology world, it was soon clear that he not only truly cared about the specific research we were doing together, but he wished to convey to his trainees an approach to science that was open, always questioning, and infinitely fun. His enthusiasm was infectious and after my training with him, despite stresses due to funding and publishing hurdles, I never regretted the path I took. My research took a sharp turn from the studies of adaptive immunity I had planned on pursuing after my fellowship with Bill to a life long quest to understand the wonders of the mast cell, a relatively rare innate immune cell. This came about because Bill’s curiosity and expectation of the unexpected allowed him to view, in retrospect, a rather mundane observation we made together involving a non-physiological transformed mast cell line as something that might be really interesting. I have never forgotten that lesson: Look at the data with an eye on the big picture. Sometimes the unexpected is more interesting than predicted results. His example in this regard was incredibly important when as an independent investigator a mistake in mouse sex determination led to unexpected and very confusing data. Yet, these data ultimately revealed a role for mast cells in male-specific protection in experimental autoimmune encephalomyelitis, the mouse model of multiple sclerosis. Bill’s influence in immunology is far-reaching and will continue to be felt as those of us who train our own students and post-doctoral fellows pass on his wisdom and approach to scientific research.

## Introduction

In January of 1983, I arrived at the Laboratory of Immunology, NIAID, NIH, to work as a post-doctoral fellow with Bill Paul. Like most scientists at the time, I was very enamored by the burgeoning revolution in molecular biology and hoped to gain expertise in gene cloning and expression analysis in the context of the very strong cellular immunology environment of Bill’s laboratory. As Bill and I discussed projects, it became clear that we needed a better way to study IL-4, a cytokine then referred to as B cell stimulatory factor-1 or BSF-1. This molecule had been recently identified by Maureen Howard and Bill as an “activity” in phorbol ester-stimulated EL4 T cell lymphoma supernatants that induced B cell proliferation (1). Only by cloning the gene encoding this molecule and having the ability to express reasonably large amounts of pure protein could we accurately determine its regulation and range of biological activities.

The task was daunting for a number of reasons: the enzymes available at the time to carry out reverse transcription were inefficient and made the synthesis of a full-length cDNA a challenge. In addition, our ability to screen for IL-4 activity was dependent on a cumbersome B cell co-stimulatory assay in which purified resting B cells are co-incubated with anti-IgD and a source of IL-4. But in consultation with Ron Germain, our resident expert in all things related to genes, we came up with a plan. I would isolate mRNA from activated EL4 T cells, size fractionate the nucleic acid, subject each fraction to *in vitro* translation using *Xenopus laevis* frog eggs, and test the protein in the B cell co-stimulatory assay. Positive fractions would be used to create a cDNA library. I ordered a colony of frogs, harvested eggs, injected RNA fractions then incubated the eggs overnight, added the egg supernatants to purified low density B cells that were co-stimulated with anti-IgD, and finally measured proliferation using a ^3^H-thymidine incorporation assay (2). After seemingly endless negative results (and embarrassing to me, multiple weekly meetings with no good data to present to Bill), one fraction showed activity and this was used as a template for a cDNA library. Unfortunately our hopeful results coincided with two reports that the gene encoding IL-4 had been identified (3, 4). Given the promise of our cDNA library, I quickly identified a clone. The race was on to determine what regulates the expression of IL-4 in normal T cells.

## Serendipitous Discovery # 1: Not all T Cells Express IL-4 but Mast Cells Do

Surprisingly, with the exception of EL-4 cells, none of the long-term T cell lines in the Laboratory of Immunology were positive in our Northern blot analyses, thereby suggesting that there are either unique T cell activation requirements for IL-4 expression and/or there is selectivity in the types of T cells that can express IL-4. Indeed, both of these possibilities turned out to be true. Not long afterward, Mossman and Coffman published their seminal paper revealing the existence of distinct CD4^+^ T helper (Th) cell subsets based on cytokine-producing potential and showed that there is a reciprocal expression pattern of IL-4 and IFN-γ in Th2 and Th1 cells, respectively (5). Subsequent studies have shown that the cytokine microenvironment of a naive CD4^+^ T cell undergoing priming dictates its initial differentiation fate [reviewed in Ref. (6)]. Although frustrating, the lack of an IL-4 response in T cell lines prompted me to take advantage of the unique access to the plethora of biological materials available at the NIH. I canvased other laboratories and collected multiple cell lines representing many distinct lineages and screened them for IL-4 mRNA. Only a subset of transformed and IL-3-dependent mast cell lines was positive.

### A Paradigm Shift in Thinking About Mast Cells’ Contributions to Health and Disease

This discovery was published in *Cell* in 1987 (7) and while in retrospect the study was extremely limited and descriptive, Bill immediately recognized the importance of the observation. At the time, studies in mast cell research were largely dictated by adherence to an old paradigm. That is, mast cell activation, mediated solely through FcεR1 cross-linking, elicits the local and immediate release of preformed pro-inflammatory mediators contained in granules. These include lysosome enzymes such as β-hexoseaminidase and cathepsin, biogenic amines such as histamine and mast cell-specific proteases, for example, tryptase and chymase, many of which are involved in eliciting the allergic response. The finding that mast cells expressed cytokines, particularly IL-4, initiated a paradigm shift. Not only could mast cells participate in the effector phase of allergic responses but given they could possibly express this cytokine at low levels without activation, these cells have the potential to directly drive IgE production by B cells.

This accidental discovery of IL-4 production in an unexpected cell type was just the beginning of a massive shift in our ideas about mast cells in health and disease, ideas that had changed only incrementally since the discovery of these cells in late 1800s (8). Subsequent studies by Marshall Plaut, Robert Seder, and Achsah Keegan in Bill’s laboratory not only demonstrated that IL-4 production is induced in activated non-transformed lines after IgE receptor cross-linking, but that activated mast cells are also a source of other cytokines, both in culture and *in vivo* (9–11). They also revealed that IL-3 priming significantly increases cytokine production by IgE-stimulated mast cells (12). Since the 1990s, there has been an explosion of data revealing both protective and pathologic roles for mast cells heralding in a new age in mast cell biology [reviewed in Ref. (13)]. Many IgE-independent modes of mast cell activation have since been described. Furthermore, both human and rodent mast cells [foreskin-derived or bone marrow-derived mast cell (BMMC) lines] express a surprisingly large number of cytokines and chemokines under multiple activation conditions. *In vivo*, mast cells have ultimately been shown to affect the outcome of many infections, autoimmune diseases and even cancer. Unexpectedly perhaps, in view of the large amount of evidence that support a pro-inflammatory role, it is now clear that mast cells also have regulatory functions and can suppress damaging immune responses.

### Mast Cell-Deficient Mice: A Key to Deciphering *In Vivo* Contributions of Mast Cells

But this re-imagining of a more widespread role of mast cells was not without controversy. Indeed, a paper published in 2011 by Hans Rodewald and colleagues (14), as well as subsequent work by this group (15) called into question the many reports demonstrating the contributions of mast cells in IgE-independent diseases. This controversy arose in part because there are still no perfect mast cell-deficient mice, although some have fewer non-mast cell defects, thus are arguably better and easier to work with.

One of the earliest descriptions of mast cell-deficient mice came in 1973 by Kitamura and colleagues (16). These mice, designated (*Kit*^W/Wv^), are the result of a cross between mice with two distinct naturally occurring mutations, *W* and *W^v^*, in *Kit*, a gene encoding c-kit, the stem cell factor receptor. Unlike most hematopoietic cells that require c-kit signaling only in early development, mast cells depend on strong and sustained c-kit signals for their development and long-term survival. WBB6 *Kit*^W/Wv^ (WB *Kit*^W/+^ X C57BL/6 *Kit*^Wv/+^)F1 mice exhibit an 80–90% reduction in c-kit signaling. While this reduced level of activity is sufficient to support the differentiation of most hematopoietic cells, mast cell development is profoundly affected. These mice are also infertile, anemic, neutropenic, have loss of melanocyte pigment production, and show defects in intestinal mobility.

Despite these issues, *Kit*^W/Wv^ mice became the gold standard for *in vivo* mast cell function studies for a period of time. Mast cells can be selectively reconstituted by systemic or local transfer of wild-type BMMCs. If a phenotype is altered in *Kit*^W/Wv^ mice and reconstitution restores it to a wild-type state yet fails to correct the inherent anemia or neutropenia, the phenotype is designated as mast cell-dependent. Mast cells were subsequently implicated in asthma, experimental autoimmune encephalomyelitis (EAE), the mouse model of multiple sclerosis (MS), arthritis, bullous pemphigus and wound healing, intestinal nematode expulsion, and protection from bacterial infections and protection from animal venoms using *Kit*^W/Wv^ mice [reviewed in Ref. (17)].

Other mice with distinct mutations in *Kit*, such as *Kit* ^W-sh/W-sh^ mice, have also enjoyed relatively widespread use because unlike *Kit*^W/Wv^ they are on a pure C57BL/6 background, are fertile and are not anemic (18). However, these mice exhibit neutrophilia as well as increased numbers of mast cell precursors and basophils. To circumvent the problems associated with *Kit* mutations, a variety of *Kit*-independent mast cell-deficient mice have now been generated. Because a selective mast cell-specific promoter has not been identified, the approach has relied on Cre recombinase expression under the control of mast cell gene “associated” promoters. Some examples: in *Cpa3*^Cre+/−^ mice, the so-called “Cre-master” mice, in which high Cre recombinase expression is driven by the *Carboxypeptidase 3* (*Cpa3*) promoter, both mast cell and basophil populations are deleted due to Cre recombinase toxicity (14). *Mast cell protease 5* (*Mcpt-5*)*-Cre* mice were crossed to *R-DTA^fl/fl^* mice resulting in diphtheria toxin produced only by Cre-expressing cells (19). These mice lose peritoneal and ear mast cells and >90% of abdominal and back skin mast cells. However, mucosal mast cells are less affected. *Cpa3-Cre; Mcl-1^fl/fl^* mice were generated by crossing *Cpa3-Cre* mice with mice containing a floxed *Myeloid leukemia sequence 1* gene and exhibit a 92–100% reduction of mast cells in all sites tested with the exception of the spleen (20). They also are anemic, have neutrophilia, and show a dramatic reduction in basophils in the bone marrow and blood.

### Mast Cells Amplify Central Nervous System (CNS) Autoimmune Disease in Female C57BL/6 and SJL Mice

Our laboratory has exclusively used the *Kit*^W/Wv^ mouse to interrogate the role of mast cells in EAE, a rodent model of multiple sclerosis (MS). MS is an autoimmune demyelinating disorder that develops when myelin-reactive Th1 and Th17 cells gain access to the brain and spinal cord through the normally restrictive blood–brain barrier (BBB) (21). Here, they orchestrate inflammatory damage to the nerve-insulating myelin sheath and the nerve axons. The loss of proper nerve conduction leads to neurological dysfunction that can range from muscle weakness and spasm to loss of motor function and cognitive defects. The most common course of disease is relapsing-remitting MS in which symptoms are intermittent. It is still unclear why MS patients generate pathogenic self-reactive myelin-specific T cells; thus, this autoreactive immune response must be recapitulated in mice by active immunization with myelin, myelin-derived peptides or through adoptive transfer of myelin-specific T cells from immunized mice that are expanded in culture under Th1- or Th17-polarizing conditions (22). Not all mouse strains are susceptible to disease, but MOG_35–55_-immunized C57BL/6 and PLP_139–151_-immunized SJL mice are commonly used as models of chronic and relapsing-remitting disease, respectively.

### Why Mast Cells in EAE/MS?

Our original studies in EAE were prompted by many reports consistent with mast cell involvement in disease. Mast cells are most often associated with blood vessels and nerves and are present in the brain, where they are most numerous in thalamus and hippocampus (23, 24). In addition to their ability to express many mediators including TNF, IL-6, and IL-1β, that promote the pathogenic immune response in MS and EAE, mast cells can also directly provoke demyelination *in vitro* suggesting a potential direct action on myelinated nerves (25). Mast cells are present in the demyelinating lesions of MS patients as are transcripts encoding the mast cell-specific protease, tryptase, as well as histamine and FcεRI (26). Tryptase and histamine are also detected in the cerebral spinal fluid of some patients (27, 28). Drugs that block mast cell degranulation (e.g., proxicromil), or deplete mast cell granules (e.g., cyproheptadine, a serotonin receptor antagonist) inhibit EAE as does hydroxyzine, a histamine receptor antagonist (29, 30).

### Mast Cells Amplify Disease Severity in EAE

In initial experiments, we observed that female *Kit*^W/Wv^ mice on the C57BL/6 and SJL backgrounds exhibit attenuated disease, a phenotype that is associated with decreased inflammatory cell infiltration to the spinal cord and brain. Selective restoration of the meningeal mast cell population *via* BMMC reconstitution is sufficient to restore wild-type disease severity and immune cell influx to the CNS (31, 32). These data indicate that the densely distributed mast cells normally residing in the meninges, a tripartite tissue that surrounds the brain and spinal cord, may be the most relevant population in EAE and MS. In the recent past, the meninges were viewed as merely physical protection for the brain and spinal cord and structures that enclosed the cerebrospinal fluid. This concept has dramatically changed, however, due to several recent discoveries: (a) lymphatic vessels are present in the meninges and provide a passageway for CNS-derived cells and molecules to access the draining deep cervical lymph nodes (33, 34); (b) T cells normally transit through the meninges as part of normal immunosurveillance of infectious microbes that threaten the CNS (35, 36); and (c) many innate immune cells, including macrophages, dendritic cells, and Type 1, 2, and 3 innate lymphoid cells, are permanent residents of these tissues, suggesting this is an immune barrier site analogous to the skin, gut, and airway mucosa (37–39). Mast cells are relatively prevalent in the dura mater, the outermost layer of the meninges, and in the pia mater, the meningeal layer that lies directly on the brain and spinal cord parenchyma. Of note, mast cells have established roles in regulating vascular permeability in peripheral tissues and in the pia mater are found in close proximity to blood vessels that transition to become the restrictive BBB vasculature. Mast cells are activated within a day of active and passive disease induction and express several mediators including IL-1β, TNF, histamine, matrix metalloproteases (MMPs), CXCL1, and CXCL2 that collectively amplify inflammation and disease severity (40, 41). Among their actions, mast cells contribute to neutrophil recruitment to the meninges and CNS. This neutrophil influx is required for altering BBB integrity and lesion initiation (42, 43). MMPs likely also affect BBB integrity by acting at the glia limitans to degrade the extracellular matrix, a function assigned to mast cells in a model of stroke (44). It has been proposed that meningeal inflammation regulated by mast cells initiates disease by allowing immune cell access to the CNS (37).

Among the most surprising actions of mast cells is their ability to “license” T cells for encephalitogenicity. Primed myelin-specific T cells are not inherently pathogenic but acquire this ability during transit from the secondary lymphoid organs to the CNS. For example, genes that assist in transendothelial migration are induced in T cells post-priming as they transit through the lungs (45). T cells in the meninges can be reactivated by myelin-bearing antigen-presenting cells (36), and it is here that T cells acquire the ability to produce GM-CSF, a cytokine essential for EAE initiation (46–48). In the CNS, GM-CSF^+^ myelin-reactive T cells recruit CCR2^+^ monocytes, the major participants in myelin destruction (49). Using an adoptive transfer model of EAE, we demonstrated that T cell-mast cell cross talk in the meninges is crucial for T cell pathogenicity (50). As a result of these interactions, mast cells express IL-1β, which acts on T cells to elicit GM-CSF. In the absence of mast cells or if mast cells are unable to express IL-1β, GM-CSF production is reduced, as is EAE severity.

It is still unclear how this cellular cross talk is initiated, although there are reports of mast cell-T cell interactions through mast cell MHC class II expression (51, 52). Others have shown that direct interactions between for example, OX40/O40L, trigger both mast cell and T cell activation suggesting a contact-dependent mechanism mediates this cross-activation (53). Finally, a recent report describes mast cell–T cell interactions promote increases in T regulatory (Treg) cell numbers in the lung draining lymph nodes in a model of allergic inflammation (54). Mast cell-T cell co-culture experiments demonstrated that mast cell-derived IL-2 was critical for this Treg cell expansion.

## Serendipitous Discovery #2: A Context-Dependent Role for Mast Cells in EAE: Sex Matters

Until recently, all of our studies to interrogate the pathologic role of mast cells were performed using female mice. This was particularly relevant in the SJL strain because male SJL mice develop little or no disease. However, an incident of inaccurate sex determination in young mice resulted in our accidental analysis of a cohort of wild-type and *Kit*^W/Wv^ males. Although it took some time to sort out, we observed that the *Kit* mutation, rather than protecting as it does in females, causes significantly worse disease in males. This unintentional finding ultimately led to surprising insight into the cellular and molecular basis of sex-dimorphic EAE susceptibility.

### Sex-Dependent Protection in EAE

Considerable efforts have been made to understand sex-dependent EAE differences in SJL mice because they provide a model of the profound differences in MS susceptibility that exist in humans where females show a threefold to fourfold higher incidence than men (22, 55–57). Several studies have demonstrated that protection in SJL males is not due to a lack of an anti-myelin response but rather to qualitatively distinct T cell responses: whereas females generate a pathogenic Th17 cell response, a non-harmful Th2 response dominates in males (58, 59).

We observed that male SJL *Kit*^W/Wv^ mice generate a Th17 anti-myelin response consistent with their clinical disease (60). Mast cell reconstitution does not restore protection in *Kit* mutant males indicating these cells are not sufficient for protection and that another c-kit^+^ cell is likely involved. Indeed, further analysis of these mice revealed an additional c-kit-dependent phenotype. Type 2 innate lymphoid cells (ILC2s) express c-kit and are also in deficit in *Kit*^W/Wv^ mice. ILC2s are CD45^+^, Lineage^−^IL-7Rα^+^ innate immune cells. They are distinguished from other members of the ILC family including ILC1s and ILC3s based on their expression of Th2 cell lineage determining transcription factors (GATA3^high^ and RORα^+^), ST2, the IL-33 receptor, and their production of Th2 cytokines. This was of interest because ILC2s are established players in immunity to parasites and allergic disease, where their expression of IL-13 is essential for robust Th2 responses (61–64). Thus, our observations suggested the possibility that the lack of ILC2s in *Kit*^W/Wv^ males prevented the development of the Th2-dominated response characteristic of male wild-type mice.

These data also raised the possibility that the Th17-dominated response in females is due to absent or dysfunctional ILC2s. Yet female SJL mice have similar steady state populations in the multiple tissues analyzed (bone marrow, lymph nodes, brain, spinal cord, meninges), and there is no difference in the response of wild-type male vs. female-derived ILC2s when provided with activating factors such as IL-33, IL-2, and IL-7 (65). However, there are sex-determined differences in the expression of activating factors, including IL-33. Upon immunization males express significantly higher levels of IL-33 mRNA in the lymph nodes, meninges, brain, and spinal cord. IL-33 is considered the most potent ILC2 activating factor (66), and the importance of this cytokine in disease protection was verified by experiments demonstrating that IL-33 treatment of females prior to disease induction prevents EAE. Importantly, treatment at peak disease reverses clinical symptoms. In both cases, ILC2s are activated and even an established Th17 response shifts to one that is Th2-dominated. Anti-IL-33 treatment of males blocks ILC2 activation and renders the mice susceptible to EAE (65).

### Mast Cells Are Activated to Express IL-33 Upon Immunization

Mast cells are one important source of this cytokine *in vivo* (65). IL-33 mRNA and protein production by mast cells can be detected in the meninges after disease induction. Furthermore, mast cell-deficient males show a significantly reduced IL-33 response upon immunization when compared to wild-type males and BMMC reconstitution partially restores this response. These data have led to a model in which male *Kit*^W/Wv^ mice fail to generate a Th2 response because they lack both an important source of IL-33, mast cells, as well as the IL-33 responder population, ILC2s.

### Testosterone-Induced IL-33 Elicits the Male-Specific ILC2-Dependent Protective Pathway

This model explains the inability to restore protection to susceptible *Kit*^W/Wv^ males with mast cells alone. But what accounts for the male-specific expression of IL-33? Testosterone was a likely candidate. This sex hormone is found at sevenfold to eightfold higher levels in adult males than females, and is associated with male-protection (57, 67). MS susceptibility in men increases with the normal age-related decline in testosterone levels, and limited clinical studies have shown treatment of male patients improves cognitive symptoms and gray matter atrophy (68, 69). In mice, testosterone treatment of females attenuates the pathogenic T cell response and reduces disease. Likewise, testosterone blockade using the androgen receptor (AR) antagonist flutamide confers susceptibility to males (70–72).

**Figure 1:**
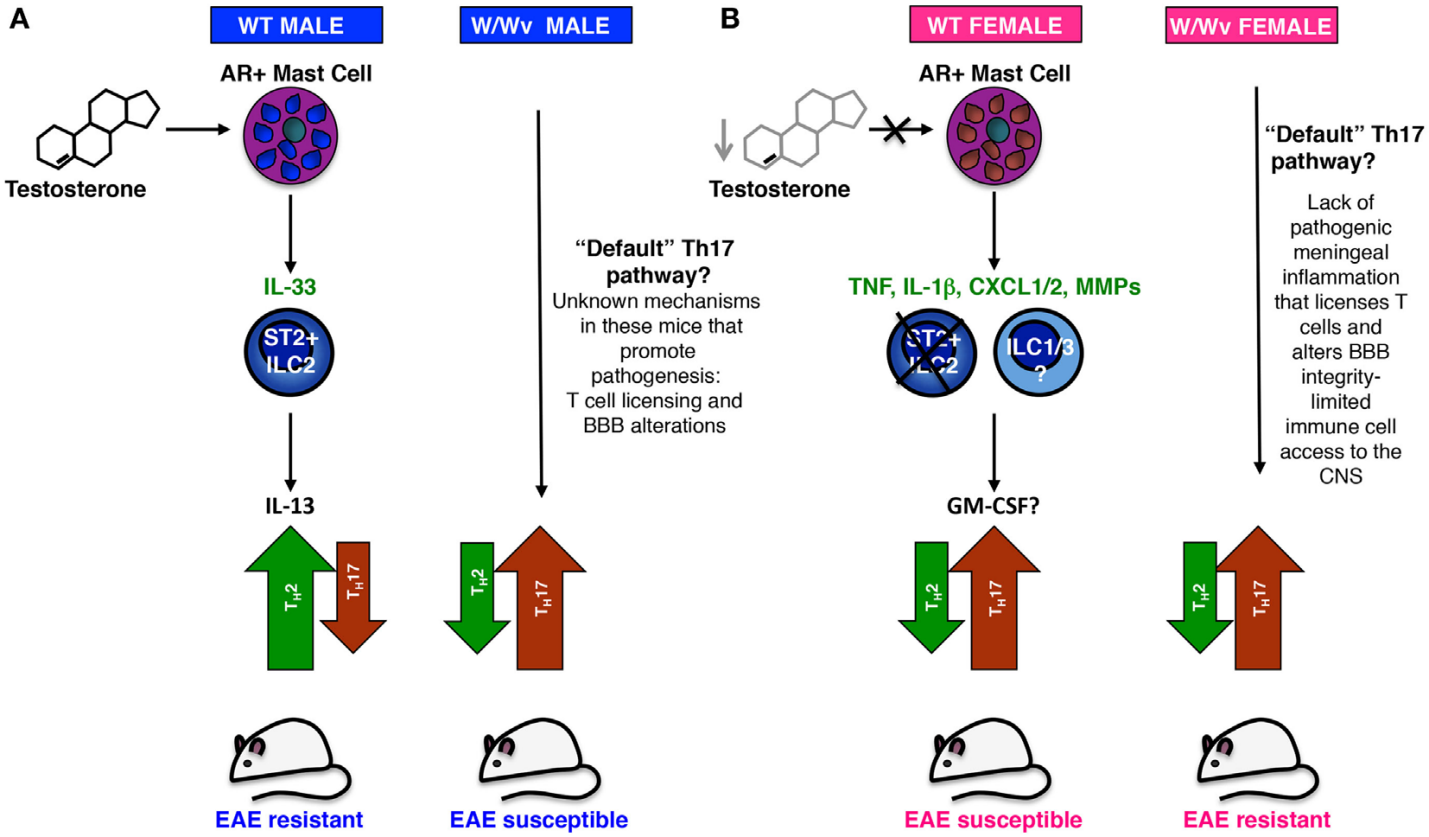
A model of sex-dimorphic T helper (Th) responses in experimental autoimmune encephalomyelitis (EAE) informed by studies in *Kit*^W/Wv^ mice. **(A)** Testosterone-dependent IL-33 production in androgen receptor^+^ (AR^+^) mast cells promotes a non-pathogenic Th2 anti-myelin response in PLP_139–151_ immunized wild-type SJL males. Early IL-33 production by mast cells (and perhaps other AR^+^) cells activates ST2^+^ innate lymphoid cells (ILC2s), which in turn express IL-13, a cytokine that polarizes the response to one that is Th2-dominant. This Th2 polarization appears to take place during priming in the secondary lymphoid organs and is likely maintained in Th2 effector cells by resident ILC2s in the meninges and Central Nervous System (CNS). Testosterone potentially acts in two ways: (1) acute increases in systemic testosterone directly activate mast cells, and perhaps other AR^+^ cells, resulting in increased IL-33 expression; (2) long-term testosterone exposure may also exert effects on the *Il33* chromatin landscape in mast cells, enabling higher potential for activation-induced expression. In the absence of IL-33-producing mast cells and ILC2s, a major but not exclusive IL-33 target cell, male *Kit*^W/Wv^ mice cannot generate a strong Th2 response and “default” to a pathogenic Th17 response. In addition to anti-myelin-specific Th17 cells, unknown mechanisms promote inflammatory cell influx to the CNS and promote disease susceptibility in these mice. **(B)** Immunized wild-type females “default” to a Th17 response because they lack sufficient testosterone to elicit the IL-33–ILC2–Th2 pathway. Low testosterone may fail to acutely induce IL-33, but may also affect the *Il33* chromatin landscape, lessening the potential for mast cell IL-33 expression. Upon activation female-derived mast cells express an alternative set of more pro-inflammatory effector molecules. IL-1β-producing mast cells “license” these T cells as they transit through the meninges by eliciting GM-CSF production and enhancing encephalitogenicity. Inflammatory cell influx to the CNS is facilitated by mast cell TNF, CXCL1/2, and matrix metalloprotease (MMP) production that recruits neutrophils and degrades the extracellular matrix, altering blood–brain barrier (BBB) integrity. Increased ILC1 and ILC3 activity in females may also facilitate meningeal inflammation and immune cell infiltration to the CNS (73). Resistant female *Kit*^W/Wv^ mice also generate a Th17 response, but in the absence of meningeal inflammation and T cell licensing, driven by mast cell-derived TNF, IL-1β, CXCL1/2, and MMPs, these cells have only limited access to the CNS parenchyma.

Both male- and female-derived peritoneal mast cells as well as BMMCs express the AR (65). However, testosterone induces IL-33 protein and mRNA expression only in male-derived BMMCs. This male-specific expression pattern was also evident with other modes of activation. Stimulation with heat killed *Mycobacterium* (Mtb) or IgE receptor cross-linking induced a relatively robust *Il33* response in male- but not female-derived cells. Taken together, we propose that testosterone induces a cascade of events that lead to the expression of mast cell IL-33, activation of ILC2s, and priming of Th2 responses (Figure 1). It is notable that immunized males show increases in serum testosterone over time, with levels peaking at ~ day 13 post-immunization. We speculate that (a) inflammation enhances the male hormonal milieu, which in turn further promotes a shift to Th2-mediated protection, and (b) females do not express the threshold level of testosterone needed to activate this pathway.

### The Ever-Evolving View of Mast Cells—We Must Leave the Paradigms Behind

There are several implications of these data in addition to the obvious possibilities for new therapeutic approaches to neuro-inflammatory diseases. First, they further demonstrate that mast cells respond in a context-dependent way. While this concept is not new when considering distinct tissue-specific actions of mast cells, we show that the hormonal context can radically alter outcomes of mast cell activation in cells derived from the same tissues. Indeed, in addition to sex-specific responses by meningeal mast cells in immunized mice, there are distinctions in BMMC responses in culture. This is strikingly illustrated by the fact that female-derived BMMCs do not express appreciable IL-33 even when stimulated with Mtb or through IgE receptor cross-linking (65). Rather these modes of activation induce *Tnf* and *Il1b*, genes that are not as highly expressed in male-derived mast cells. It is likely that in addition to acute influences on mast cell activation, the hormonal environment shapes the overall potential for gene expression in these cells by altering chromatin accessibility. The directive from the NIH director that sex must be considered as a biological variable has not come too soon.

Our findings add to the growing evidence that mast cells can serve protective roles in some settings. Evidence showing that mast cell–Treg cell interactions can be important in limiting inflammation also continues to accumulate. Mast cells appear to act downstream of Treg cells in an allograft tolerance model in which mast cells are required for prolonged survival. It is proposed that IL-9 production by Tregs activates local mast cells to produce IL-10 to limit rejection (74). In a papain-induced model of allergic inflammation, mast cells act upstream of Tregs (75). In this scenario, IL-33, presumably passively released after protease damage of lung epithelial cells, elicits IL-2 production by mast cells. IL-2 promotes Treg cell expansion and limits the damaging effector response mediated by eosinophils. In view of these studies and given the reported protective IL-33-dependent role of a subpopulation of ST2^+^ Tregs in a model of inflammatory bowel disease (76), it will be important to understand whether these and other ST2^+^ cells are targets of this mast cell produced cytokine in EAE/MS. Mast cells limit inflammatory damage in a Treg-independent manner as well. Not only do mast cell proteases degrade animal venoms and can decrease the pathological responses associated with envenomation (77), IL-10 and IL-2 produced by mast cells limit chronic inflammation in models of contact sensitivity (78, 79) and in a graft-versus-host disease model where mast cell-derived IL-10 is required for prolonging graft survival (80).

### Are Mast Cells Really the Master Cell?

As alluded to above, results generated using *Kit*^W/Wv^ mice have been called into question because they are often not replicated when *Kit* ^W-sh/W-sh^ or *Kit*-independent mast cell-deficient mice are used [discussed in beautiful detail in Ref. (17)]. A stunning example is the multitude of papers using Cre-master mice to demonstrate that mast cells are dispensable in many settings where mast cells were previously shown to make a contribution [reviewed in Ref. (15)]. The original report by Rodewald’s group showed that anaphylaxis and expansion of intestinal mast cells in a *N. brasiliensis* infection model are extinguished in *Cpa3*^Cre+/−^ mice, supporting the validity of using these mice to assess mast cell contributions in responses in which IgE-activated mast cells are the major effector cells (14). However, unlike previous (but not all) EAE studies by us and by others using *Kit*^W/Wv^ mice suggesting mast cells exacerbate disease (81), *Cpa3*^Cre/+^ mice are fully susceptible to EAE. The reasons for these differences are still unclear, but there are several possibilities: Mast cells provide an accessory function that can amplify or lessen a response mediated by activated T and B cells. In cases where strong T or B cells are induced, the more subtle contributions of mast cells may be masked. Evidence that altering the EAE disease induction protocols affects the ability to assign a mast cell contribution comes from multiple laboratories using the same *Kit*^W/Wv^ mice (81). Of note, the strong disease induction conditions used in the Cre-master mouse study (corroborated by high morbidity in all groups) also support this alternative interpretation of the data. Age of mice and environment, including differences in microbiota, are also variables that may affect disease severity.

So what do we make of all the data that comes from *Kit*^W/Wv^ mice? The dramatically different mast cell functions revealed by our analyses of male and female *Kit*^W/Wv^ mice in EAE confirm that, under the right experimental conditions, *Kit* mutant knock in mice are valid tools to delineate the role of mast cells and other c-kit^+^ cells in disease models. While we still need more selective ways to genetically deplete mast cells, the data generated from studies with *Kit*^W/Wv^ mice should not be discarded out of hand: in females, the lack of mast cells resulted in reduced clinical disease, which is restored to wild-type severity with reconstitution. Although disease scoring is too often subjective, more objective assessments revealed the alteration of several mast cell-dependent pathways that amplify inflammation. These include meningeal mast cell activation, neutrophil influx to the meninges, BBB breach, inflammatory cell influx to the CNS, mast cell IL-1β expression in the meninges, and acquisition of T cell GM-CSF production. Importantly, our use of male *Kit*^W/Wv^ mice revealed a pathway that could not have been easily identified in other *Kit*-independent mast cell-deficient mice. Mast cell reconstitution failed to confer protection to *Kit*^W/Wv^ males, indicating mast cells alone cannot restore the male-specific wild-type phenotype. Thus the system worked, as it should. Indeed, these experiments allowed us to identify the deficit in c-kit^+^ ILC2s in *Kit*^W/Wv^ mice and to assign them as additional critical players in male-specific protection. The role of a c-kit^+^ pro-inflammatory ILC3 population in EAE exacerbations was also revealed using these mice (38). It is tempting to speculate that c-kit^+^ ILCs may contribute to other functions assigned solely to mast cells using *Kit*^W/Wv^ mice. That is, the lack of both mast cells and ILCs in *Kit*-dependent mast cell-deficient mice may explain some of the discrepancies observed in studies using *Kit*-independent mast cell-deficient mice in which ILC populations are likely unaffected.

As alluded to earlier, mast cells have the potential to influence many if not most biological processes in humans due to their widespread distribution in most tissues, their proximity to blood vessels, the seemingly endless variety of effector molecules they can produce, and their ability to interact with both immune and non-immune cells. Indeed, in a review by Rodewald and Feyerbend it was stated, “There is arguably no second cell type in the immune system as powerfully equipped with a large array of chemically diverse and highly potent compounds” (15). Not surprisingly, soon after the realization that mast cells can act outside the realm of allergy the experimental dam broke so to speak, leading to many studies over the years showing mast cells modulate processes far beyond the innate and adaptive immune responses that dictate the outcomes of autoimmunity, cancer, infection and neuroinflammation. Among the perhaps unexpected activities of mast cells are roles in vascular disease (82), angiogenesis and tissue remodeling (83, 84), diabetic wound healing (85), migraine headaches (86), anxiety (87), metabolic syndromes (88), fertility (89, 90), and development of mammary glands (91).

The challenges ahead are many. First, it is important to ultimately delineate the underlying reasons for the conflicting data derived from various experimental models. Second, the observed strain and sex variations in mast cell activity defined in mice indicate that many new paradigms that arose based on studies in one mouse strain or sex must be revisited to take these variables into account. Third, there is likely to be similar and more extensive mast cell heterogeneity in humans. Uncovering these differences will be a daunting task. An ultimate goal may be to target these cells in disease therapy, but in some settings, we will need to understand their actions in each individual context in order to make decisions about whether blocking or enhancing their activation is desirable. Only by keeping our eyes on the big picture, we will continue to gain greater insight into the biology of these amazing cells, cells which I have made my life’s passion, all because of Bill Paul.

## Author Contributions

The author confirms being the sole contributor of this work and approved it for publication.

## Conflict of Interest Statement

The author declares that the research was conducted in the absence of any commercial or financial relationships that could be construed as a potential conflict of interest.
